# Character recognition competition for street view shop signs

**DOI:** 10.1093/nsr/nwad141

**Published:** 2023-05-19

**Authors:** Jingqun Tang, Weidong Du, Bin Wang, Wenyang Zhou, Shuqi Mei, Tao Xue, Xing Xu, Hai Zhang

**Affiliations:** Shanghai ByteDance Technology Co., Ltd, China; School of Control Science and Engineering, Zhejiang University, China; School of Computer Science and Technology, University of Science and Technology of China, China; School of Computer Science and Technology, Tsinghua Univeristy, China; Shenzhen Tencent Technology Co., Ltd, China; Shenzhen Tencent Technology Co., Ltd, China; School of Computer Science and Engineering, University of Electronic Science and Technology of China, China; Pazhou Laboratory (Huangpu), China; School of Mathematics, Northwest University, China; Pazhou Laboratory (Huangpu), China

## Abstract

This paper presents the inaugural character recognition competition for street view shop signs, including the associated tasks, datasets, participating teams, the winning team's solution, and justification for the award.

## INTRODUCTION

Store signboards provide important information in street view images, and character recognition in natural scenes is an important research direction in computer vision. Street view store signboard character recognition technology, a combination of the two, is widely used in map navigation and recommendation, smart city planning analysis, business value analysis in commercial districts and other practical fields, with high research and commercial values.

Each competing team needs to develop a shop signboard recognition and analysis scheme based on street view photos and labels provided by the event organizer, to achieve shop signboard detection, layout analysis and shop name recognition in natural street view photos. The recognition scenes of the algorithm are the natural street scenes, the target objects of recognition are the store signboards and their text contents and the recognition environment is restricted to images from daytime. The input of the algorithm is a street view image, and the output is the location of the store sign in the image (not all the signboards in the image), and the name of the store in the store sign (not all the text lines in the store sign). The goal of the algorithm is to improve the signboard detection accuracy of the algorithm and the accuracy of the detected store names in the test set.

### Challenge

This competition has the following challenges.

1. *Complex tasks.* This task consists of three tasks: signboard detection, text box detection and text recognition.
2. *Complex scene.* The recognition scene is a streetscape image with complex information. At the same time, an image may contain multiple signboards and non-signboards.

### Competition details

#### Dataset

This competition provides three data sets, namely, ‘Store sign and store name data’, ‘Signboard detection data’ and ‘Signboard OCR data’. The format of the evaluation set shall be consistent with the ‘Store sign and store name data’. The following is an introduction to each data set.

##### Store sign and store name data.

**Figure 1.**
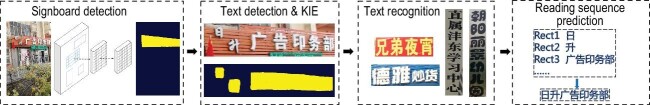
The algorithm pipeline. KIE represents key information extraction.

This dataset contains street view images. Each image may contain multiple storefronts. Each storefront contains multiple signboards, some of which are non-store signboards, and some of which are store signboards. There is the possibility that there may not be a store sign in the picture. The dataset labels indicate the valid quadrilateral box of the store signboard in each figure and its corresponding store name. (Valid store signboards are defined as signboards with complete and clear shapes, without any significant occlusion, and containing the specific name of the store.) The training set size is around 5000, and the testing set size is around 500. In the test set, the store names contain at least two Chinese characters and do not contain English characters or special characters. The tag file is in txt format. Each row of the tag content is formatted as $x_1, y_1, x_2, y_2, x_3, y_3, x_4, y_4, store\_name$. Here *x*_1_, …, *y*_4_ are the coordinates of the four footpoints, arranged clockwise, of the shop sign frame; $store\_name$ is the shop name corresponding to the shop signboard. Samples from the dataset are shown in Fig. 1 within the online supplementary material.

##### Signboard detection data.

This dataset contains street view images. Each image may contain multiple storefronts. The image of this dataset is the same as that of store sign and store name data. Note that some store signboards from this dataset may not be valid, that is, there may not be corresponding store names for these store signboards in the first dataset. There may also be no store signs in the picture. This dataset label marks the quadrangle boxes of all the signboards in each figure.

The amount of training data is consistent with the shop sign and shop name data. The tag file is in txt format. Each row of the tag content is *x*_1_, *y*_1_, *x*_2_, *y*_2_, *x*_3_, *y*_3_, *x*_4_, *y*_4_, 0/1. Here *x*_1_, …, *y*_4_ are the coordinates of the four footpoints, arranged clockwise, of the signboard frame, 0 represents the non-store signboards and 1 represents the store signs. Samples from this dataset are shown in Fig. 2 within the online supplementary material. In the images, the green boxes mark the shop signs, and the red boxes mark the subsidiary signs.

##### Signboard OCR data.

This dataset is the close-up data of all the signboards, which is cropped from the above street view data. Each picture contains multiple lines of text. The dataset label indicates the position and content of all text in each photo. The data size is 14 331 images. The tag file is in txt format. Each row of the tag content is *x*_1_, *y*_1_, *x*_2_, *y*_2_, *x*_3_, *y*_3_, *x*_4_, *y*_4_, *text*, where *x*_1_, …, *y*_4_ are the four foot coordinates of the *text* and *text* is the text content. Samples from this dataset are shown in Fig. 3 within the online supplementary material.

#### Evaluation metric

In order to support domestic machine learning development, the competition uses the Jittor [1] framework to provide the baseline code of the competition. Jittor is a high-performance deep learning framework based on instant compilation and meta operators. The entire framework integrates powerful Op compilers and tuners with instant compilation to generate customized high-performance code for the neural network model. Jittor also contains a rich high-performance model library, covering image recognition, detection, segmentation, generation, differentiable rendering, geometry learning, reinforcement learning, etc.

The evaluation metrics of the competition are the accuracy and recall rates of store signboard identification. Only when the location of the detection frame of the shop signboard is accurate and the identification of the shop name line in the signboard is correct, the identification is defined to be accurate. Theformula for calculating the recall rate of store sign recognition is the ratio of the number of correctly recognized store signs to the true number of store signs. The calculation formula of the accuracy rate of store sign recognition is the ratio of the number of correctly recognized store signs to the total number of recognized store signs. The final score is the F-score of the accuracy rate and recall rate, calculated as 2 × *accuracy* × *recall*/(*accuracy* + *recall*). For example, in the test set, there are a total of 10 000 store signs, and the contestants identified 12 000 signs, of which 9000 were correctly identified. Therefore, the accuracy rate is 9000 divided by 12 000, which is 0.75. The recall rate is equal to 9000 divided by 10 000, which is 0.9. So the final score is 2 × 0.9 × 0.75/(0.9 + 0.75), which is 0.81.

## ALGORITHM

As shown in Fig. 1, our team uses a multi-stage algorithm, which is divided into four main stages: signboard detection, signboard text detection and KIE, text recognition, and reading sequence prediction.

### Signboard detection

In the signboard detection stage, combined with raw annotation data, the labeled data are reprocessed into three categories: valid store signboards, invalid store signboards and non-store signboards. It is because we need to distinguish the invalid shop signs in the signboards, but the raw annotations only indicate whether or not they are store signboards. Since the ground truth is a quadrilateral region, we choose the method of instance segmentation instead of object detection to segment the mask of the store signboard, then select the smallest external quadrilateral of the mask and perform perspective transformation on the quadrilateral for perspective text correction, and finally get the corrected signboard region. Based on Mask-RCNN, the model structure of instance segmentation is improved in three aspects: (i) adding a deformable convolutional network (DCN) to enhance the feature extraction capability, (ii) using four vertices as keypoints and adding keypoint regression branches to improve model performance in a multi-task learning approach, (iii) in addition to the usual data augmentation methods, copy-and-paste and random perspective transformation methods are employed.

### Signboard text detection and KIE

In the stage of text detection and signboard text extraction phase, the two tasks are combined for end-to-end training due to the commonality of text features. The pipeline of the model is shown in Fig. 4 within the online supplementary material. First, the model uses a two-stage detector to detect the location of the text and extract both the positional embedding and the image embedding of the text. A graph neural network is then used to integrate multi-modal features and distinguish which belong to store signboard texts. The end-to-end training paradigm, which is inspired by former work [2], allows for more powerful and effective features to be learned, thereby improving the accuracy of both tasks simultaneously. In addition, we use a reinforcement learning-based method called BoxDQN [3], which adjusts the shape of the text box to find the optimal box for the recognition model, thus improving the accuracy of end-to-end recognition.

### Text recognition

In the text recognition phase, we judge the direction of the text based on the aspect ratio of the text box, and then train two text recognition models for horizontal and vertical text, respectively. The baseline of the text recognition model we use is SAR [4]. Based on it, we make some improvements: (1) upgrading the backbone to VIT [5], (2) changing the unidirectional LSTM to bidirectional LSTM, which integrates LSTM and two-dimensional (2D) attentional features in parallel, and provides richer contextual information, (3) using multiple self-supervised training and (4) adding center loss to boost the discrimination of morphological close word recognition features. Two self-supervised pre-training methods that contribute significantly are described below. The first one is a pre-training method based on sequential contrast learning [6], as shown in Fig. 5 within the online supplementary material. In detail, we preform some augmentation on the text image to get a positive sample pair, and then select another text image as a negative sample pair. The model extracts the sequence features using an encoder, and then learns from the positive and negative sample pairs based on the sequence. The second one is masking pre-training based on stroke information and semantic context. As shown in Fig. 6 within the online supplementary material, it is an improvement based on MAE [7], where different masks are set for stroke features and semantic features to predict and learn the masks. In this way, the mural information and textual information of the text can be modeled simultaneously to improve the feature representation of the model. Using these two different self-supervised pre-training methods, we can obtain an extractor with strong modeling ability, thus improving the performance of text recognition.

### Reading sequence prediction

In the reading sequence prediction stage, since the store signboard text does not always consist of only one text block, it needs to be merged according to the relative position of the text blocks. We simply follow the logical relationships for text box reading sequence prediction without any use of a neural network.

### Details of the models

The signboard detection model is based on Mask-RCNN with a Resnet-50 DCN as the backbone. In addition to box regression and classification heads, we add a keypoint regression branch. The joint model for the signboard text detection and key information extraction tasks includes a two-stage detector like Faster-RCNN, a position tokenizer and a GNN-based multi-modal feature aggregator. As for the recognition model, the backbone is a VIT-small model followed by a bidirectional encoder that integrates LSTM and 2D attentional features in parallel.

## RESULTS

Overall, our approaches can be found in Table 1 within the online supplementary material. In this competition, we incorporate three techniques that have become popular in recent years: multimodal feature fusion, large-scale self-supervised training and the transformer-based large model. In addition, depending on the properties of the data provided, we add some illuminating methods such as BoxDQN based on reinforcement learning and text retification, which achieve promising performance.

### Comparisons with other methods

#### Comparisons with other teams

The second place team uses a horizontal box detector for signboard detection, which does not take into account the multi-directionality and perspective distortion of the signboard. In the text recognition phase, they do not use a self-supervised approach for pre-training, but they have a large amount of extra annotated data, which results in a recognition performance comparable to ours. In the KIE and reading order prediction phases, they use a GNN model, which would have consumed a lot of time. In contrast, we do not use a separate model, so our time consumption is superior.

#### Comparisons with existing popular approaches

The baseline model we chose for the competition is the SOTA model, which is also popular. As shown in Table 1 within the online supplementary material, our approach makes several modifications over the industry’s usual baseline approach and improves the performance of the model considerably. For example, the self-supervised pre-training strategy for text recognition delivers a gain of 3.2.

### Award-winning reason

There are many challenges in the street view store signboard recognition task, such as the various modes and content of store signboards, geometric distortion and truncation in image shooting, and limited training samples. The baseline team from ByteDance, with its profound accumulation in the OCR field for many years, has better analyzed the difficulties and proposed a reasonable algorithm framework according to the characteristics of the competition, and won first place in the competition. The overall framework divides the competition task into four stages: signboard detection, signboard text detection, text recognition and reading sequence prediction. The main highlights are ‘signboard text detection and key information extraction’, ‘mutli-modal modelling & GNN’, ‘text self-supervised pre-training’ and ‘VIT model’. This method effectively integrates multi-dimensional information such as image features and text position distribution for multi-modal recognition and reasoning. In the case of limited samples, self-supervised pre-training and data augmentation methods are used to significantly improve the performance in a short period of time.

Finally, the baseline team won first place among 14 teams with an F-score of 0.6672, an oral defense score of 92.86 and a combined score of 97.11. With a reasonable design, this framework can be quickly implemented and applied in industry, and can play a good role in restoring the real world and building more realistic maps.

## FUTURE WORK

With the in-depth development of deep learning, many breakthroughs have been made in the field of computer vision. The understanding of images has been deepened from simple detection, segmentation and classification tasks to scene understanding, multi-modal recognition, relational reasoning and end-to-end image understanding.

In the evolution of computer vision research on text recognition in street view store signboard recognition, the following trends have emerged. (1) Semi-supervised or unsupervised learning-based large-scale pre-training is adopted to learn and understand the implicit relationship and internal connection of road data, and provides general pre-training representation for down-streaming perception tasks. This method can reduce the amount of finely labeled data, increase the generalization capability of the model and decrease the cost of research and development. (2) Multi-dimensional feature-based multi-modal multi-task object relationship analysis is employed to increase the recognition accuracy. (3) Scene recognition and quality recognition of the object is used to filter the invalid data. (4) End-to-end image understanding is also developing rapidly. The technology will eventually develop into a real-world map auto generation technology that combines target content, scene analysis and location restoration, and will play an important role in navigation and intelligent recommendation, smart city planning and analysis, and automatic driving.

## Supplementary Material

nwad141_Supplemental_File
