# Supplementary material for: Character recognition competition for street view shop signs
Source: Natl Sci Rev. 2023 May 19;10(6):nwad141. doi: 10.1093/nsr/nwad141 (PMC10281498; doi:10.1093/nsr/nwad141)
Supplement: nwad141_Supplemental_File [file nwad141_supplemental_file.pdf]

Appendix

1 Challenge

Some examples of the store signboard data in the competition are shown in Fig. 1, Fig. 2 and Fig. 3.

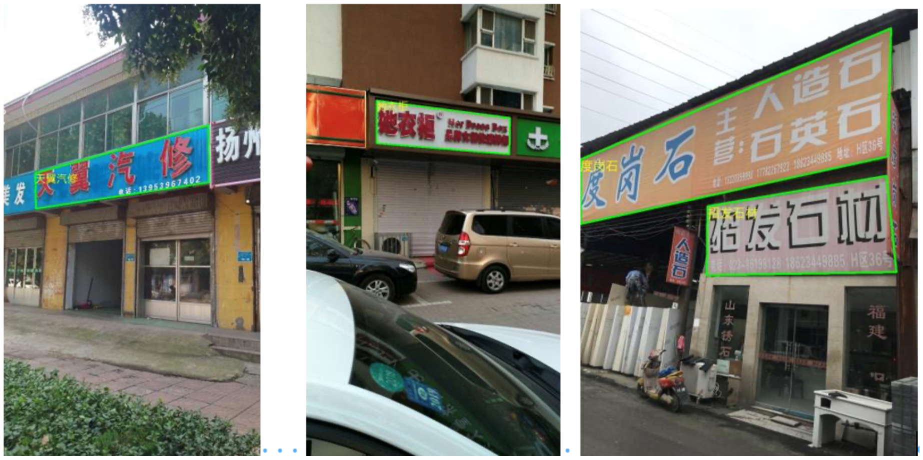

Figure 1: Data examples of store sign and store name data.

2 Algorithm

The pipeline of text detection and KIE is shown in Fig. 7. Fig. 5 and Fig. 6 show the pipeline of self-supervised pretrain for text recognition. Fig. 7 shows the pipeline of perspective text rectification. And the Tab. 1 summarizes the proposals of the first place team.

References

[1] Tang J, Qian W, Song L *et al.* Optimal boxes: Boosting end-to-end scene text recognition by adjusting annotated bounding boxes via reinforcement

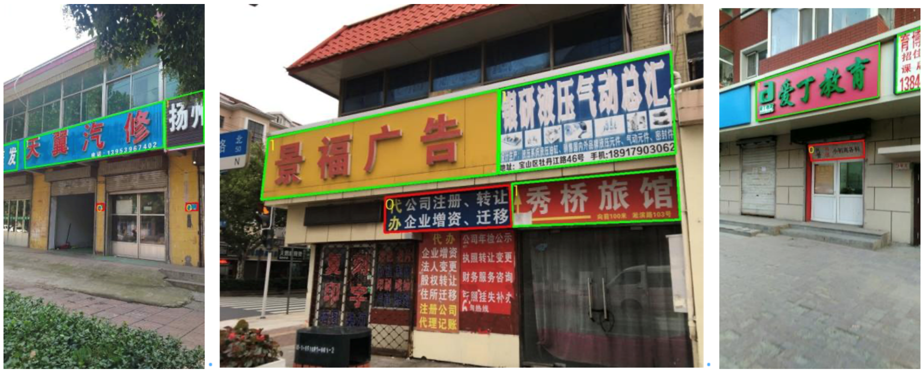

Figure 2: Data examples of signboard detection data.

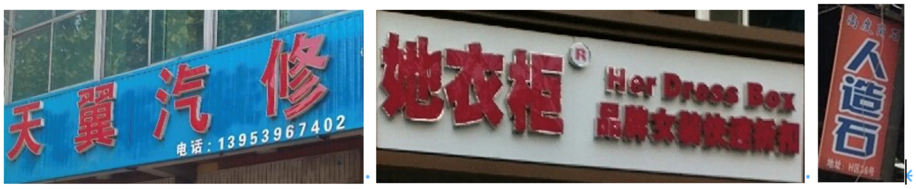

Figure 3: Data examples of signboard OCR data.

learning. Avidan S, Brostow GJ, Cissé M *et al.*, editors, *Computer Vision - ECCV 2022 - 17th European Conference, Tel Aviv, Israel, October 23-27, 2022, Proceedings, Part XXVIII*, volume 13688 of *Lecture Notes in Computer Science*, (Springer2022) 233–248.

[2] Dosovitskiy A, Beyer L, Kolesnikov A *et al.* An image is worth 16x16 words: Transformers for image recognition at scale. *9th International Conference on Learning Representations, ICLR 2021, Virtual Event, Austria, May 3-7, 2021*, (OpenReview.net2021) .

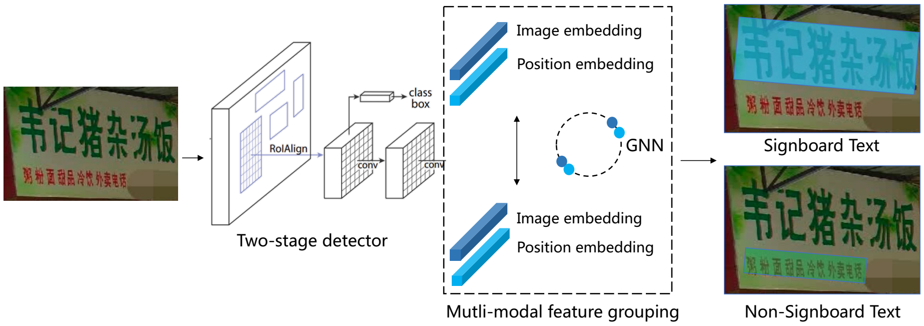

Figure 4: The end-to-end network for joint text detection and KIE tasks. The model consists of two major parts. One is a two-stage detector that detects the position of the text and extract the positional embedding and image embedding of the text. The other is a graph neural network for integrating multimodal features and distinguishing which text belongs to the store signboard.

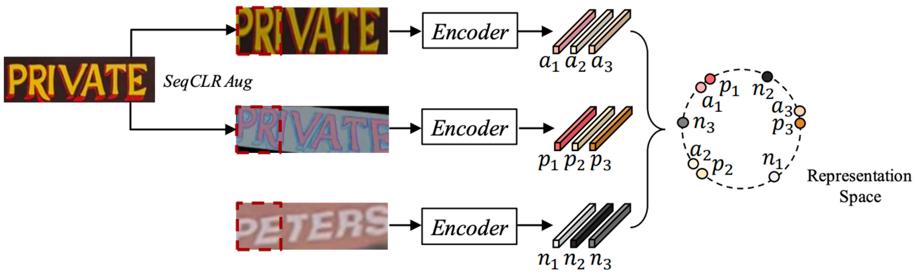

Figure 5: Sequential Contrast Learning (SCL) for boosting scene text recognition.

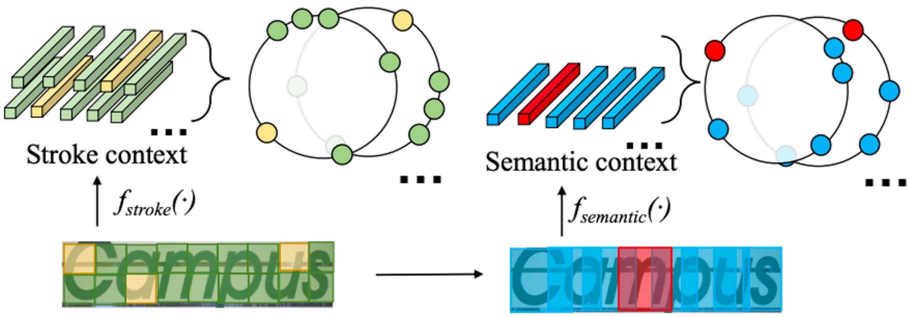

Figure 6: Stoke and semantic context-based masked image modeling.

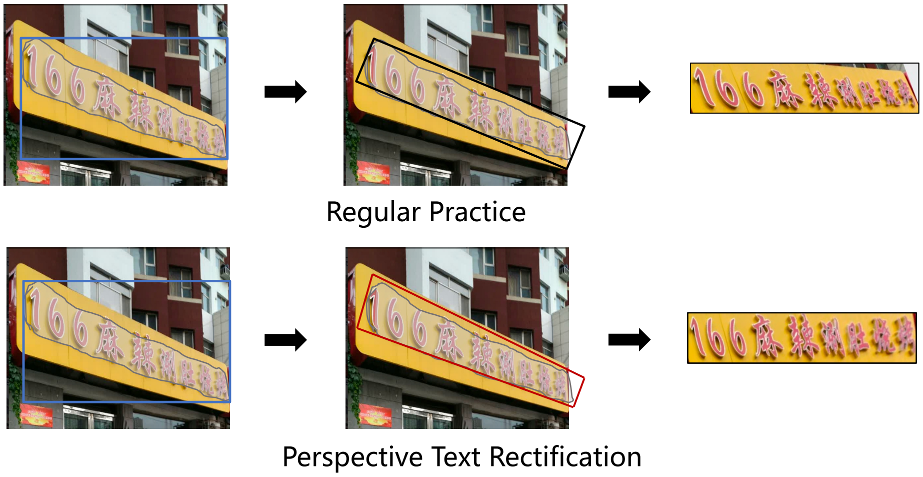

Figure 7: Perspective text rectification. Compared with the conventional method of directly intercepting the minimum enclosing rectangle, our method is to obtain the minimum enclosing quadrilateral and then carry out perspective transformation to get a more accurate text region.

Table 1: The methods that are applied in the competition and their contribution.

Method	Gain (%)
DCN	0.6
BoxDQN [1]	1.6
VIT [2] backbone	2.5
Self-supervised pre-training	3.2
Mutli-modal modelling & GNN	2.9
Perspective text rectification	2.6
Center Loss for text recognition	0.8
